# Pineal progenitors originate from a non-neural territory limited by FGF signalling

**DOI:** 10.1242/dev.171405

**Published:** 2019-11-21

**Authors:** Nicole Staudt, Florence A. Giger, Triona Fielding, James A. Hutt, Isabelle Foucher, Vicky Snowden, Agathe Hellich, Clemens Kiecker, Corinne Houart

**Affiliations:** Department for Developmental Neurobiology, Guy's Hospital Campus, King's College London, London SE1 1UL, UK

**Keywords:** Circumventricular organs, Neural plate border, Neurulation, Otx, FGF, Placode, Pineal, Endocrine, Zebrafish, Chick

## Abstract

The embryonic development of the pineal organ, a neuroendocrine gland on top of the diencephalon, remains enigmatic. Classic fate-mapping studies suggested that pineal progenitors originate from the lateral border of the anterior neural plate. We show here, using gene expression and fate mapping/lineage tracing in zebrafish, that pineal progenitors originate, at least in part, from the non-neural ectoderm. Gene expression in chick indicates that this non-neural origin of pineal progenitors is conserved in amniotes. Genetic repression of placodal, but not neural crest, cell fate results in pineal hypoplasia in zebrafish, while mis-expression of transcription factors known to specify placodal identity during gastrulation promotes the formation of ectopic pineal progenitors. We also demonstrate that fibroblast growth factors (FGFs) position the pineal progenitor domain within the non-neural border by repressing pineal fate and that the Otx transcription factors promote pinealogenesis by inhibiting this FGF activity. The non-neural origin of the pineal organ reveals an underlying similarity in the formation of the pineal and pituitary glands, and suggests that all CNS neuroendocrine organs may require a non-neural contribution to form neurosecretory cells.

## Abstract (precis)

**Highlighted Article:** Gene expression and fate mapping/lineage tracing in zebrafish reveals that the pineal organ develops from the non-neural pre-placodal ectoderm under the control of FGF signalling.

## INTRODUCTION

The pineal organ (also known as pineal gland or epiphysis cerebri), one of the circumventricular organs, is an endocrine gland located above the diencephalon in the brain of most vertebrates. The human pineal organ is about half a centimetre in length and can be found in the superior cistern, wedged in between the cerebral hemispheres. Its main function is the cyclical production of melatonin, which affects the modulation of sleep, food intake, breeding and sexual maturity in both diurnal and seasonal rhythms (Macchi and Bruce, 2004; Arendt and Skene, 2005). The pineal organ contains photoreceptors that molecularly and structurally resemble the photoreceptors of the retina. This has led to the suggestion that the pineal organ represents a phylogenetically ancient photosensitive organ that may have lost its photoreceptive function in mammals (Klein, 2004; Mano and Fukada, 2007).

Comparably little is known about the mechanisms that regulate pineal development in vertebrate embryos (Joly et al., 2007; Rath et al., 2013; Sapède and Cau, 2013; Kiecker, 2018). The homeodomain transcription factors Not1/Noto (also known as floating head, Flh, in zebrafish), Pax6, Otx2 and Bsx are required for pinealogenesis in rodents, zebrafish and frogs, and individuals with mutations in *PAX6* frequently lack the pineal organ (Masai et al., 1997; Estivill-Torrús et al., 2001; Cau and Wilson, 2003; Mitchell et al., 2003; Nishida et al., 2003; Foucher et al., 2006; Abouzeid et al., 2009; D'Autilia et al., 2010; Chatterjee et al., 2014; Khuansuwan et al., 2016; Schredelseker and Driever, 2018). Similar to the outpocketing of the optic vesicles from the ventrolateral diencephalon, the mammalian pineal organ is thought to emerge through evagination of the roof of the embryonic diencephalon (Oksche, 1965). Together with the habenular nuclei, the pineal organ forms the epithalamus, a complex that has been studied extensively as a model for asymmetric neurogenesis in the zebrafish embryo (Bianco and Wilson, 2009; Roberson and Halpern, 2018). However, how and where pineal progenitors are initially specified, and how these are shaped into the pineal organ remains unclear. Classic fate-mapping studies using quail-chick chimeras or fluorescent dye labelling in the frog *Xenopus laevis* placed the pineal primordium at the lateral edge of the anterior neural plate (Couly and Le Douarin, 1987; Eagleson and Harris, 1990).

Here, we have used a combination of molecular marker analysis, fate mapping, time-lapse analysis and genetics in zebrafish and chick to resolve the issue of the embryonic origin of pineal progenitors. We find that the pineal organ is specified during neurulation and that a large part of it originates outside of the neural plate, from the pre-placodal region (PPR), a domain of non-neural ectoderm that gives rise to placodes (Streit, 2007; Schlosser, 2014). Thus, the pineal organ is similar to other sensory and neuroendocrine structures of the vertebrate head, such as the eye and the pituitary gland (also known as hypophysis) that form with contributions from both neural and placodal tissues (Graw, 2010; Sánchez-Arrones et al., 2015). We have investigated the mechanisms that establish and restrict pineal identity within the PPR and show that (1) the fibroblast growth factor (FGF) signalling pathway functions as an antagonist of pineal identity, indicating that FGFs released from both the midbrain-hindbrain boundary and the anterior neural ridge contribute to the positioning of the anteroposterior limits of pinealogenesis; and (2) that the orthodenticle-like homeodomain transcription factors Otx1 and Otx2 promote pineal organ formation by suppressing this antagonistic FGF activity.

## RESULTS

### Non-neural origin of pineal progenitors

**Fig. 1.**
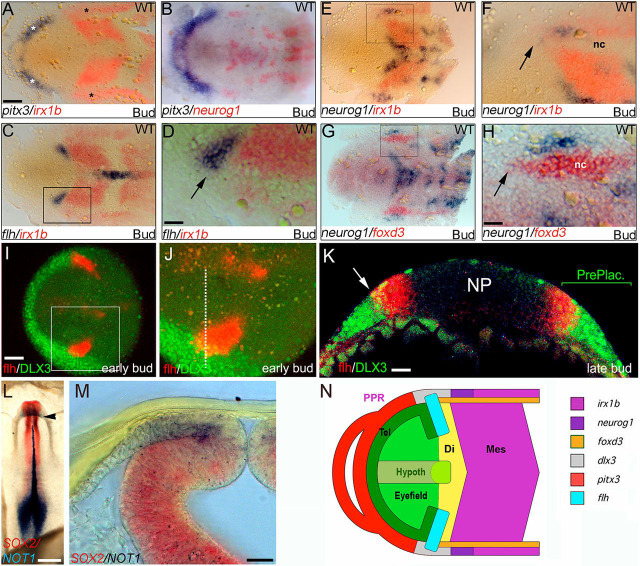
**The *flh/noto/NOT1*-expressing pineal progenitor region extends into the PPR.** (A-H) Dual-colour *in situ* hybridisation for indicated genes on bud stage zebrafish embryos (anterior is leftwards). White asterisk in A marks *pitx3*-positive anterior PPR; black asterisks mark *irx1b*-positive posterior PPR. (D,F,H) Magnified views of the boxed areas in C,E,G, respectively; arrows indicate a putative pineal progenitor region; nc marks the neural crest in F,H. (I) *In situ* hybridisation for *flh/noto* (red) and immunohistochemical detection of DLX3 (green) in an early bud stage zebrafish embryo. (J) Magnification of boxed area in I showing partial overlap between *flh/noto* and DLX3 expression. (K) Transverse section of late bud stage embryo stained as in I and J at the level of the diencephalon. Arrow indicates the overlap between *flh/noto* and DLX3-expressing cells in the PPR (PrePlac.). All zebrafish wild-type double staining experiments were performed with at least 20 embryos, two independent experiments each. Images are representative of most or all embryos for each set of markers. (L) HH8.5 chick embryo stained using dual-colour *in situ* hybridisation for *SOX2* (red) and *NOT1* (blue; anterior is upwards). Arrowhead indicates level of section in M. (M) Transverse section through the neural tube of an embryo stained as in L at the level of the posterior forebrain (arrowhead in L). There is *NOT1* staining in non-neural ectoderm overlying the *SOX2*-positive neural tube (*n*=3; two independent experiments). (N) Schematic colour-coded representation of gene expression patterns shown in A-K (Di, diencephalon; Hypoth, hypothalamus; Mes, mesencephalon; Tel, telencephalon). Scale bars: in A, 50 µm for A-C,E,G; in D,H, 15 µm for D,F,H,J; in I, 50 µm; 15 µm in K; 500 µm in L; 25 µm in M.

During an unbiased neural plate fate-mapping study in zebrafish, we observed that the diencephalic territory expressing *flh/noto –* published as the pineal progenitor territory at neurula stage (Masai et al., 1997; Cau and Wilson, 2003) – is largely fated to contribute to the thalamus, raising the question of where pineal organ precursors can be found at this stage (Staudt and Houart, 2007). As classical fate-mapping experiments had placed pineal progenitors at the border of the anterior neural plate (Couly and Le Douarin, 1987; Eagleson and Harris, 1990), we decided to characterise gene expression in this region in zebrafish in more detail. The early neural plate is surrounded by a horseshoe-shaped domain of non-neural ectoderm, the pre-placodal region (PPR), that gives rise to the cranial placodes (Streit, 2007; Schlosser, 2014). The anterior PPR (which gives rise to the adenohypophysis, the anterior part of the pituitary gland) is marked by expression of the homeobox gene *pitx3* (Fig. 1A,B), whereas the posterior PPR is marked by expression of the *iroquois*-related homeobox gene *irx1b* (Fig. 1A,C-F)*.* The most anterior part of the *irx1b*-positive PPR that is known to give rise to the trigeminal placode also expresses *neurogenin 1* (*neurog1*), which encodes a basic helix-loop-helix transcription factor involved in neurogenesis (Fig. 1B,E-H). *irx1b* is also expressed in the posterior neural plate, but the prospective neural crest that lies between the posterior neural plate and PPR, and is marked by expression of *foxd3*, is *irx1b* negative (Fig. 1F,H).

When we analysed the expression of the bona fide pineal progenitor marker *flh/noto* in relation to these different domains, we found that its territory reaches further laterally than the expression of *irx1b* in the neural plate (Fig. 1C,D), suggesting that it might extend into the neural crest and/or PPR domain. In order to test whether *flh/noto*-positive cells reside in the PPR, we performed double staining for *flh/noto* and the pan-PPR marker DLX3, using a combination of *in situ* hybridisation and antibody staining, and observed that there is indeed an overlap of the expression of these two factors in the neural plate border region (Fig. 1I-K). The chick orthologue of *flh/noto*, *NOT1*, is not expressed in the ectoderm at early neural plate stages, but it appears in the non-neural ectoderm overlying the diencephalon at around Hamburger-Hamilton (HH) stage 8, when the neural tube is starting to close (Fig. 1L,M) (Hamburger and Hamilton, 1951). This comparatively ‘late’ onset of ectodermal *NOT1* expression in the anterior neural folds of the chick embryo is consistent with what has previously been described (Stein et al., 1996). These findings show that the *flh/noto*-positive domain encompasses non-neural progenitors that are located within the PPR and that this potential non-neural contribution to pinealogenesis may be conserved in amniotes (chick). A diagram summarising the results from our gene expression mapping analysis in fish can be found in Fig. 1N.

**Fig. 2.**
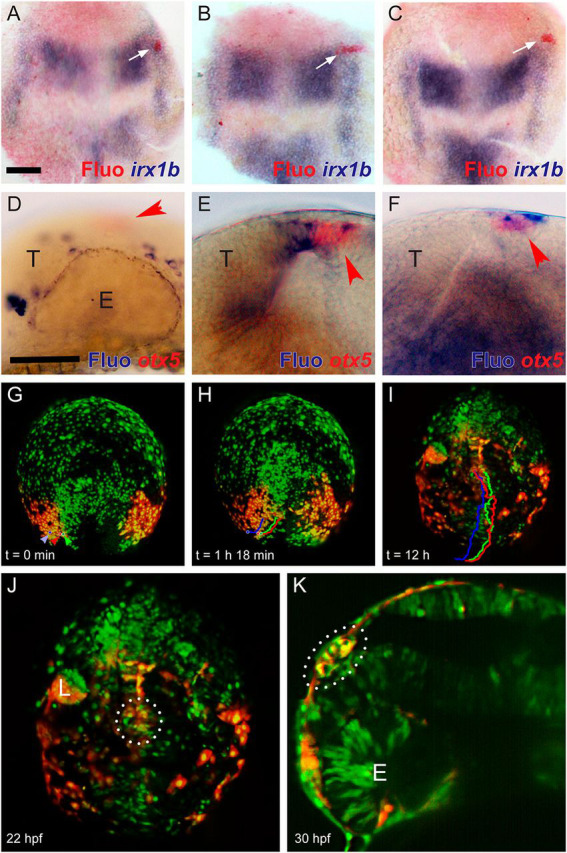
**Non-neural ectoderm contributes to the pineal organ.** (A-C) Small patches of cells (white arrows) were labelled using uncaging of fluorescein (Fluo, red) at bud stage. Embryos were fixed immediately and subjected to *in situ* hybridisation for *irx1b* (blue) to demonstrate precision of labelling (anterior is leftwards). A, *n*=6; B, *n*=4; C, *n*=7. (D-F) Lateral views (anterior is leftwards) of the diencephalon of embryos labelled as in A-C, incubated for 24 h and labelled for expression of *otx5* (red) marking the definitive pineal organ (red arrowheads). Cells labelled by uncaging (blue) in the *otx5*-positive pineal organ are labelled in E and F. D, *n*=11; E, *n*=11; E, *n*=19/23. (G-I) Sequential images of time-lapse recording of an embryo from late bud to the 26-somite stage in which Kaede-expressing cells have been converted to red fluorescence in the ectoderm flanking the diencephalon. The trajectories of three representative cells (blue, red and green arrowheads in G) are shown in red, green and blue in H,I. (J) Dorsal view showing converted cells in the pineal organ at 22 hpf. See also Movies 1 and 2. (K) Lateral view, anterior towards the left, showing converted cells in the ectoderm and pineal organ, but not in the brain proper, at 30 hpf. Four out of 12 embryos had no converted cells in the brain proper; all four of those showed converted cells in the pineal organ. E, eye; L, lens; T, telencephalon. Dotted lines in J and K outline the pineal organ. Scale bars: 50 µm; bar in A applies to A-C,G-I; bar in D applies to D-F,J,K.

In order to refine our mapping of the origin of pineal progenitors in fish, and to verify that the DLX3-positive region of the *flh/noto* domain is indeed contributing to the pineal organ, we uncaged fluorescein in the neural plate border at the level of the posterior diencephalon in transgenic Tg(*her5:eGFP*) embryos (Staudt and Houart, 2007). The accuracy of this approach was checked by fixing a few embryos minutes after uncaging and staining them for *irx1b* (Lecaudey et al., 2001). Although cells labelled within the posterior PPR and prospective neural crest domains (Fig. 2A) frequently contribute to the trigeminal placode and neural crest (Fig. 2D), and uncaging across both neural and non-neural territories in this region (Fig. 2B) leads to staining of both epithalamus and pineal cells (Fig. 2E), cells labelled in a small border domain immediately anterior to the *irx1b*-positive posterior PPR (Fig. 2C) most often ended up in the pineal organ proper, which can be labelled for the *orthodenticle*-like homeobox gene *otx5* at 24 h post fertilisation (hpf) (Fig. 2F).

To monitor how non-neural cells integrate into the pineal organ, we performed time-lapse light-sheet microscopy recordings of embryos injected with Kaede mRNA in which we photo-converted the non-neural ectoderm along the border of the anterior neural plate at the tail-bud stage (Fig. 2G-K; Movies 1 and 2) (Ando et al., 2002). In four out of 12 embryos treated in this manner, no photo-converted cells were found within the neural tube, indicating that only non-neural ectoderm had been labelled. In these four embryos, many labelled cells were found in the epidermis as expected and a few migrating neural crest cells were also labelled. Moreover, in all four of these embryos, a subset of labelled cells integrated into the dorsal-most neural keel between the six- and eight-somite stages (Fig. 2J), and contributed to the pineal organ that is morphologically distinguishable as a button-like structure on top of the diencephalon at 30 hpf (Fig. 2K). Retrospective tracing of three such cells revealed that they clearly originated within the non-neural ectoderm laterally flanking the diencephalic territory (colour-coded traces in Fig. 2H,I; Movie 2).

**Fig. 3.**
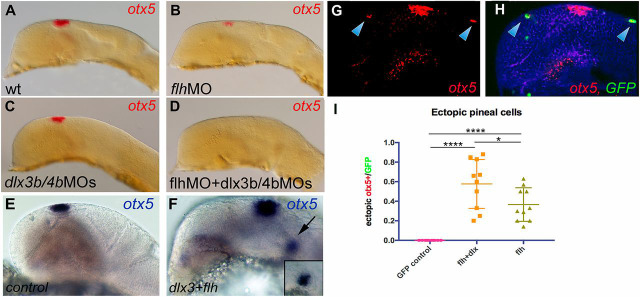
**The PPR specifier genes *dlx3b/dlx4b* in conjunction with *flh/noto* are required and sufficient for pineal progenitor specification.** (A-D) 24 hpf zebrafish embryos injected with the MOs indicated in B-D and stained for the pineal marker *otx5* in red (lateral views, anterior is leftwards). There is reduced *otx5* staining in B and C, and a complete absence in D. Scale bar: 50 µm. MO injection experiments were performed three times independently, injecting each MO or combination of MOs in parallel into 40-50 embryos from the same parents. Images are representative for each experimental condition. Fifty percent of the embryos injected with *dlx3b/4b* MOs looked as in C, the other 50% had a range of even weaker *otx5* staining. (E,F) Injection of *dlx3* and *flh* mRNAs at the one-cell stage results in ectopic induction of the pineal marker *otx5* (blue) after 24 h of incubation (*n*=21/32; control embryo shown in E; two independent experiments). Inset in F is an image focusing on the ectopically induced *otx5*-expressing cells that are marked with the arrow in F. Scale bar: 50 µm. (G,H) Lateral views of embryos injected at the one-cell stage with *hs:flh/noto* and *hs:dlx4b*, heat-shocked at shield stage and stained by *in situ* hybridisation for *otx5* (red) after 24 h of incubation. There is sparse ectopic induction of *otx5* in superficial cells (arrowheads). Scale bars: 50 µm (bar in A applies to A-D; bar in E applies to E-H). (I) Quantification of *otx5* induction in cells injected with *hs:gfp* alone, with *hs:flh/noto* and *hs:dlx4b*, or with *hs:flh/noto* alone (two independent experiments; six embryos were selected for statistical analysis; data are mean±s.d.). *P*<0.0001 (GFP versus flh+dlx), *P*<0.0001 (GFP versus flh), *P*=0.041 (flh+dlx versus flh).

Our gene expression and fate-mapping studies indicate that pineal precursors emerge from the PPR. Esterberg and Fritz previously demonstrated that PPR identity can be blocked in zebrafish by simultaneous morpholino (MO)-mediated knockdown of *dlx3b* and *dlx4b*, which encode the homeodomain transcription factors that are required for the specification of the neural plate border region (Esterberg and Fritz, 2009). Thus, we decided to use these *dlx3b/4b* MOs to functionally assess a requirement for the PPR in pineal organ formation. The pineal organ was reduced in size in embryos injected with MOs against *flh/noto* (Fig. 3A,B), and a similar reduction was found in *dlx3b/4b* double morphants (Fig. 3C). This result could indicate either that non-PPR tissues such as the neural crest or neural tissue proper also contribute to the pineal organ, resulting in partial pinealogenesis in the absence of the PPR, or they could simply be due to a hypomorphic effect of the MOs. However, the formation of the pineal organ is completely repressed by simultaneous knockdown of *flh/noto* and *dlx3b/4b* (or by injection of *dlx3b/4b* MOs into *flh* mutant embryos), leading to a complete absence of the gland in such triple loss-of-function embryos, suggesting a synergistic role of *flh/noto* and PPR identity in pinealogenesis (Fig. 3D).

Conversely, combined ectopic expression by mRNA injection of *dlx3b* and *flh/noto* at the one-cell stage resulted in cells expressing the pineal organ marker *otx5* in ectopic locations (*n*=21/32; Fig. 3E,F), whereas neither *dlx3b* nor *flh/noto* alone had this effect (*n*=34 for *dlx3b* and *n*=51 for *flh/noto*). Ectopic *otx5*-positive cells are only found anterior to the midbrain-hindbrain boundary, indicating a posterior restriction of competence for pineal precursor induction. The overall morphology of the neural tube in embryos injected with *dlx3b* and/or *flh/noto* tends to be highly abnormal, presumably owing to early effects of these factors on gastrulation and neurulation. Thus, we generated a conditional expression system by cloning *dlx4b* and *flh/noto* into heat shock-inducible plasmids that drive the expression of green fluorescent protein (GFP)-tagged versions of these two genes (Lewis et al., 2004). Embryos injected with these constructs at the one-cell stage and heat-shocked at 37°C at late gastrula/early neurula stage (75-90% epiboly) display sparse ectopic induction of *otx5* in ectopic *flh/noto*-expressing cells and a higher rate of ectopic *otx5* expression in double *dlx4b/flh-*expressing cells (Fig. 3G-I). Consistent with a non-neural origin of pineal progenitors, we observed that ectopic *otx5*-positive cells were almost exclusively found in non-neural ectodermal cells (and occasionally among migrating neural crest cells).

Two distinct cell populations, the PPR and the neural crest, form in close proximity in the neural plate border region of the posterior diencephalon, and the expression of the pineal progenitor marker *flh/noto* appears to overlap with both of these to some extent (Fig. 1; Patthey and Gunhaga, 2011, Groves and LaBonne, 2014). The overlap of *flh/noto* expression with *dlx3* (Fig. 1I-K) and our MO knockdown experiments targeting *dlx3b/dlx4b* function (Fig. 3) have demonstrated that the PPR makes an essential contribution to pinealogenesis. To test whether the neural crest also contributes to the pineal organ, we blocked neural crest formation in zebrafish using MOs against the neural crest specifier genes *foxd3* and *sox10* (Whitlock et al., 2005), or injected the *foxd3* MO into *sox10* mutants. Neither individual nor combinatorial loss of *foxd3* and *sox10* function affected pineal organ formation, indicating that pineal precursors are not derived from the neural crest (Fig. S1).

Taken together, our experiments so far demonstrate that pineal precursors originate (at least in part) from the PPR, and that pre-placodal identity is required for pineal organ formation. Ablation experiments targeting precursors in late gastrula and neurula embryos indicated that pineal precursors are specified by neurula stage (Houart et al., 1998; Staudt and Houart, 2007). This evidence for early specification is supported by our *dlx3b/4b* loss-of-function experiments, as these two genes are only transiently expressed in the PPR at late gastrula and early neurula stages (Esterberg and Fritz, 2009), and by the conditional *dlx4b/flh* gain-of-function experiments in which ectopic pineal cell identity could specifically be induced during gastrulation.

### Pineal organ formation requires Otx gene function cell non-autonomously

**Fig. 4.**
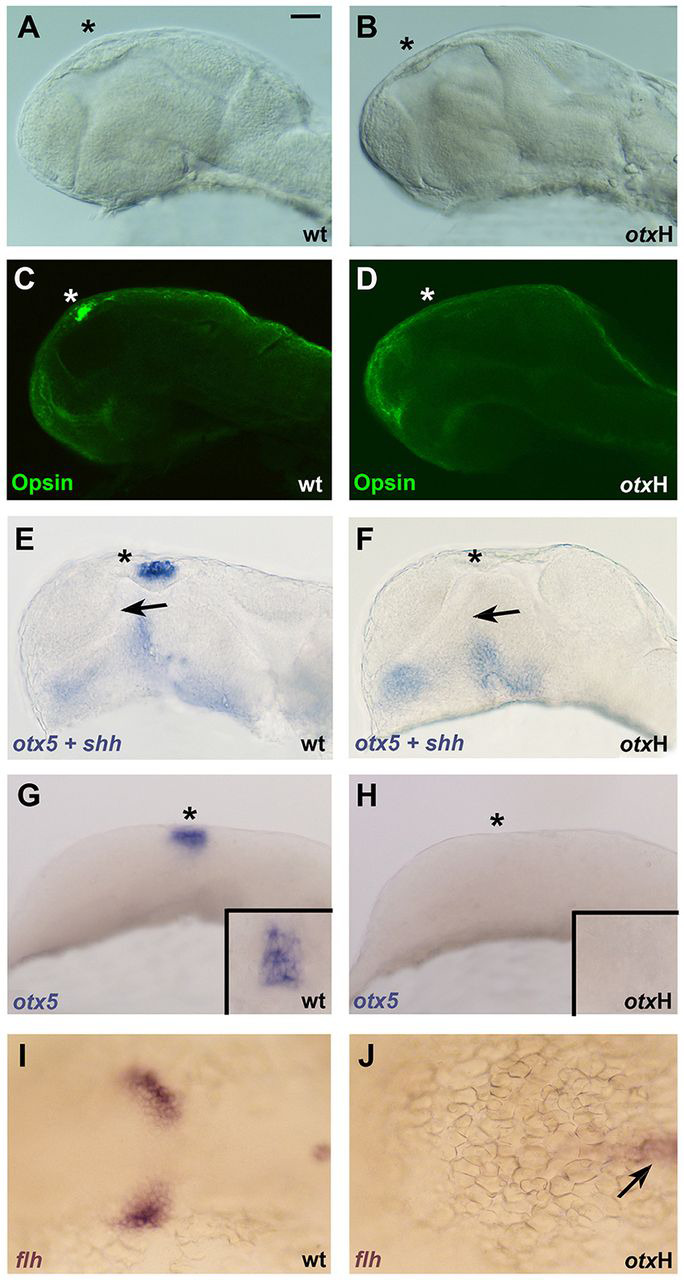
**Otx function is required for pinealogenesis.** Wild-type zebrafish embryos are shown in A,C,E,G,I and *otxH* MO-injected embryos in B,D,F,H,J (anterior is leftwards, lateral views are shown in A-H, dorsal views in I,J). Asterisks mark the location of the pineal organ in A-H. (A,B) The pineal organ is morphologically reduced in *otxH* embryos at 48 hpf. (C,D) Immunohistochemical staining for opsin at 48 hpf; staining is absent from the pineal area in D. (E,F) Double *in situ* hybridisation for *otx5* and *shh* (both in blue) at 24 hpf. *otx5*-positive pineal progenitors are absent in F. Arrows indicate the zona limitans intrathalamica; this signalling centre is reduced in F. (G,H) *In situ* hybridisation for *otx5* at the eight-somite stage, including dorsal views of the pineal region shown in the insets. (I,J) *In situ* hybridisation for *flh/noto* at bud stage; *flh/noto*-positive pineal progenitors are absent in J (arrow in J indicates persistent *flh/noto* staining in notochord). Each marker was tested in two independent experiments, comparing at least 30 control and *otxH* MO-injected embryos for each marker and stage. Images are representative of all analysed embryos. Scale bar: 50 µm.

In previous studies, we observed that injection of MOs against *otx1* and *otx2* (*otxH*) resulted in embryos that lack the pineal organ (Foucher et al., 2006; Scholpp et al., 2007). Conditional ablation in the mouse had previously demonstrated a cell-autonomous requirement for *Otx2* in pinealocyte development at later stages of development (Nishida et al., 2003). However, *otxH* morphant zebrafish display a more profound pineal defect, with a complete absence of a morphologically recognisable pineal organ (Fig. 4A,B) and of photoreceptor differentiation (assessed by expression of the photoreceptor marker α-opsin; Fig. 4C,D) at 48 hpf, as well as absence of expression of the pan-pineal marker *otx5* at 24 hpf (Fig. 4E,F) and at the eight-somite stage (Fig. 4G,H). The complete absence of *flh* expression from the anterior neural plate of *otxH* morphants at late gastrula (bud) stage indicates that Otx gene function is already required for the earliest steps of pineal precursor specification (Fig. 4I,J).

**Fig. 5.**
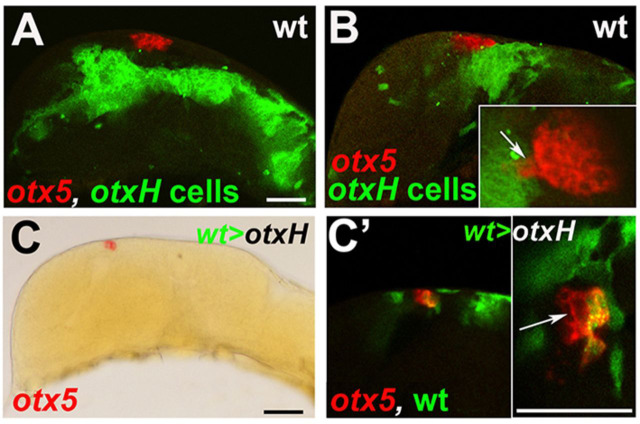
**Wild-type cells can rescue pineal progenitor specification cell non-autonomously in *otxH* morphants.** (A,B) Representative fluorescent images of wild-type zebrafish embryos containing cells grafted from an *otxH* embryo (green) and stained by *in situ* hybridisation for *otx5* (red) at 24 hpf (lateral views, anterior is leftwards). Inset in B shows a dorsal view of the pineal region in more detail. *otx5* is expressed in *otxH* cells that are adjacent to wild-type cells (arrow). Two independent experiments were performed grafting 13 and 19 embryos, respectively. (C) Bright-field image of an *otxH* embryo containing grafted wild-type cells and stained by *in situ* hybridisation for *otx5* (red) at 24 hpf (lateral view, anterior is leftwards). (C′) Fluorescent images of the pineal region of the embryo shown in C in more detail; grafted wild-type cells are green. There is non-cell-autonomous rescue of *otx5* expression (arrow). Inset in C′ shows a magnified dorsal view of the *otx5*-positive pineal organ in C′. Two independent experiments were performed grafting 17 and 14 embryos: 15/17 and 11/14 showed cell-non-autonomous pineal rescue. Scale bars: 50 µm in A,C′; 50 µm in insets (bar in the inset in C′ applies to the insets in both B and C′).

To test whether pineal precursors require Otx function cell-autonomously, we transplanted *otxH* cells into wild-type embryos. Transplanted cells had a strong tendency to avoid the roof of the forebrain and the pineal organ proper (Fig. 5A,B). *otx5*-expressing cells are occasionally seen among *otxH*-injected cells, suggesting that the requirement for Otx gene function is not strictly cell autonomous (inset in Fig. 5B). Wild-type cells transplanted into *otxH* morphant embryos showed better integration and were more evenly spread throughout the embryo. Such mosaic embryos typically displayed formation of a slightly smaller and/or somewhat disorganised pineal organ in the correct location (Fig. 5C,C′). *otxH* cells contributed to the pineal organ in these embryos, indicating that wild-type cells rescue pineal precursor specification cell non-autonomously (Fig. 5C′).

### FGF signalling represses pineal organ specification

**Fig. 6.**
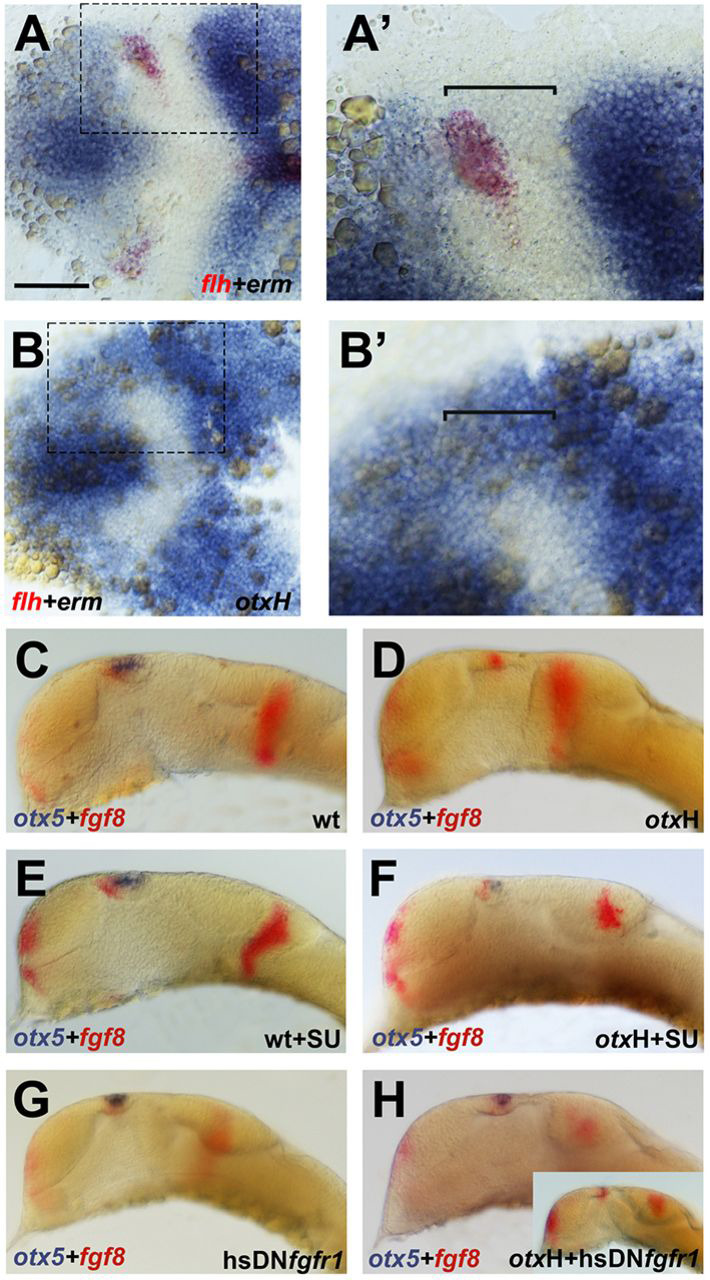
**Upregulation of FGF signalling activity in the neural plate border region is responsible for the absence of pineal progenitors in *otxH* morphant embryos.** (A-B′) Representative images of dual colour *in situ* hybridisation for *erm* (blue) and *flh* (red) in wild-type (*n*=29; A,A′) and *otxH* (*n*=32; B,B′) bud stage zebrafish embryos; anterior is leftwards. (A′,B′) The boxed neural plate border region in A,B in more detail. (C-H) Wild-type (C,E,G) and *otxH* morphant (D,F,H) zebrafish embryos stained for *fgf8* (red) and *otx5* (blue) at 24 hpf (anterior is leftwards, dorsal towards the top; C,D, *n*=18). Embryos in E,F were treated with the FGF inhibitor SU5402 (*n*=25 and *n*=22, respectively). Embryos in G,H are transgenic for a heat shock-inducible dominant-negative FGF receptor and were heat-shocked at shield stage (*n*=19 and *n*=22, respectively). *otx5*-positive pineal progenitors are rescued by pharmacological inhibition of FGF signalling in F and by heat shock-induced receptor inhibition in H (compare with D). Inset in H shows a sibling from the Tg(*hsp:dnfgfr1*) cross that did not show rescue and is therefore likely to be from the 25% of offspring that are expected to be negative for the transgene. Results are based on two independent experiments. Scale bar: 50 µm.

The pineal anlage is enlarged in *mbl* embryos, suggesting that WNT signalling promotes pineal precursor specification (Fig. S1) (Heisenberg et al., 1996; Masai et al., 1997). However, neither experimental up- nor downregulation of WNT signalling was able to rescue pinealogenesis in *otxH* morphants, suggesting that the pineal *otxH* phenotype is not related to WNT. Expression of *erm*, a bona fide target gene of the FGF signalling pathway, showed a significant increase in the neural plate border region of *otxH* morphants (Fig. 6A-B′), prompting us to test whether FGF inhibition can rescue pineal organ development in these morphants. We used the FGF inhibitor SU5402 at a concentration low enough not to result in severe morphological alterations of the brain and found that pinealogenesis is indeed rescued in *otxH* morphants treated with this pharmacological effector (Fig. 6C-F). Because SU5402 may interfere with signalling pathways other than the FGF pathway, we confirmed these results by blocking FGF signalling more directly using a zebrafish line carrying a heat shock-inducible dominant-negative FGF receptor transgene Tg(*hsp70:dnfgfr1a-eGFP*) (Lee et al., 2005). Heat-shock activation of this transgene at 30% epiboly (early gastrula) efficiently rescued the pineal organ in *otxH* morphants (Fig. 6G,H), whereas activation at a slightly later stage (70-75% epiboly, mid-gastrula) resulted in very few rescued cells in only 40% of the embryos. Similar to our SU5402 titration, we adjusted the duration of the heat shock in these experiments to avoid extensive brain abnormalities that would result from an ongoing strong inhibition of FGF signalling. Taken together, these results provide further evidence that pineal precursors are specified during gastrulation and demonstrate that excessive FGF signalling suppresses pinealogenesis in *otxH* morphant embryos.

**Fig. 7.**
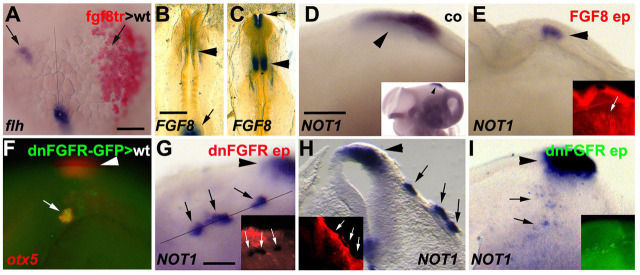
**FGF signalling antagonises pinealogenesis.** (A) Bud stage zebrafish embryo (anterior points to the top) containing transplanted *fgf8*-overexpressing cells in the right side of the neural plate (red), labelled using *in situ* hybridisation for *flh* (blue). *flh*-positive pineal progenitors are present on the right side (arrows; *n*=7/7). (B,C) Chick embryos (anterior towards the top) at HH8.5 (B) and HH9.5 (C) stained using *in situ* hybridisation for expression of *FGF8* (*n*=6; two independent experiments). Expression is present in the midbrain-hindbrain boundary in B (arrowhead; the arrow marks expression in the tailbud region). Expression is present in the anterior neural folds (arrow) and midbrain-hindbrain boundary (arrowhead) in C. (D) HH18 chick embryo stained using *in situ* hybridisation for *NOT1* (lateral view of diencephalon, anterior is rightwards). Inset shows same embryo at lower magnification. (E) Chick embryo electroporated at HH10 with FGF8 and eGFP, fixed after 36 h of incubation and stained by *in situ* hybridisation for expression of *NOT1* (blue) and by immunohistochemistry for GFP (red, inset). Arrowheads mark the pineal organ; the arrow in the inset marks the location of electroporated cells at some distance from the pineal organ. *NOT1* is downregulated in E compared with D (*n*=11/15; two independent experiments). (F) Zebrafish embryo (anterior is leftwards, dorsal towards the top) transplanted at 30% epiboly stage with heat shock-inducible *dnfgfr*-transgenic donor cells (green), heat-shocked at shield stage, fixed at 24 hpf and labelled by *in situ* hybridisation for *otx5* (red). Ectopic pineal progenitors are induced following heat shock (*n*=9/12). Arrowhead highlights *otx5*-positive pineal organ; arrow indicates ectopic *otx5*-positive cells. (G) Lateral view (anterior towards the right) of a chick embryo electroporated at HH8 with dnFGFR and eGFP, and stained after 24 h of incubation by *in situ* hybridisation for *NOT1* (blue) and by immunohistochemistry for GFP (red, inset). Arrowhead indicates *NOT1*-positive pineal organ; black and white arrows highlight ectopic *NOT1*-positive cells. (H) Oblique section of embryo along the line indicated in G. Arrowhead indicates pineal organ; arrows indicate clusters of *NOT1*-expressing cells in superficial ectoderm (*n*=6/13). (I) Lateral view of chick embryo electroporated at HH8 with dnFGFR and eGFP into the right anterior neural fold, and stained for *NOT1* after 36 h of incubation (arrowhead indicates pineal organ). Inset shows GFP fluorescence before *in situ* hybridisation. There is ectopic induction of *NOT1*-positive pineal progenitor cells in electroporated neural tube (arrows; *n*=7/27). Scale bars: 50 µm in A for A-F; in B, 500 µm for B,C; in D, 200 µm for D,E,I; 100 µm in G for G,H.

Our findings suggest that the pineal precursor domain is restricted by FGF signalling. Indeed, transplants from *fgf8*-expressing (but not from *gfp*-expressing control) donor embryos into early gastrula stage (30% epiboly) wild-type host embryos inhibited *flh/noto* expression at the late gastrula stage, demonstrating that FGF signalling is sufficient to inhibit pineal specification in fish (Fig. 7A). In chick, the first faint expression of *FGF8* in the midbrain-hindbrain boundary region is observed at HH8.5 (Fig. 7B), around the time of *NOT1* induction in the anterior neural folds (Fig. 1L). Strong expression of *FGF8* in the midbrain-hindbrain boundary and anterior neural folds is seen from around HH9 onwards (Fig. 7C). In order to test whether FGF8 also represses pineal identity in chick, we *in ovo* electroporated a *FGF8*-expressing plasmid into the prospective diencephalon of HH9/10 chick embryos. This treatment consistently resulted in downregulation or absence of *NOT1* expression after 36 h of incubation (Fig. 7D,E). Many embryos electroporated with *FGF8* showed morphological alterations that are characteristic of the diencephalon-to-midbrain transformation induced by FGF8 (Crossley et al. 1996), and only embryos that had retained a morphologically distinguishable diencephalic vesicle were included in this analysis. The competence for FGF signalling to repress pineal organ specification appears to be limited in time, as electroporation of *FGF8* into the neural tube at HH13/14 did not result in a noticeable downregulation of *NOT1* (*n*=7/7).

FGF signalling is not only sufficient, but also necessary to delimit the pineal precursor domain, as transplantation of cells from Tg(*hsp70:dnfgfr1a-eGFP*) donors into wild-type zebrafish embryos at 30% epiboly followed by heat shock at 50% epiboly and fixed at 24 hpf resulted in ectopic patches of *otx5* expression in the head ectoderm (Fig. 7F). Again, the competence for pineal induction appears to be restricted to the ectoderm anterior to the midbrain-hindbrain boundary, as posterior *dnfgfr1a*-expressing cells consistently failed to express *otx5*, which is reminiscent of the restriction observed in our *dlx4b/flh* overexpression experiments (Fig. 3F).

To test whether the requirement for FGF signalling in delimiting pineal progenitor induction is conserved in chick, we electroporated a dominant-negative FGF receptor 1 expression construct (dnFGFR) into the anterior neural folds at HH7/8 (one- to four-somite stage). Over half of the embryos electroporated at these comparably early stages die within 24 h of incubation or develop with severe neural tube defects that make the interpretation of their morphology impossible. However, in a subset of the surviving embryos, ectopic *NOT1*-expressing cells were observed in the superficial, non-neural ectoderm after 24 h of incubation (Fig. 7G,H). These non-neural clusters of ectopic *NOT1* expression were hardly ever seen after 36-48 h of incubation, suggesting that ectopic *NOT1*-positive ectodermal cells may be eliminated or expelled from embryos. At these later stages, only a few cells that show weak ectopic expression of *NOT1* were observed within the electroporated area of the neural tube of a subset of embryos (Fig. 7I). Taken together, FGF signalling antagonises pineal progenitor induction in both anamniotes (zebrafish) and amniotes (chick). In both systems, most ectopically induced pineal progenitors are found in the non-neural ectoderm, consistent with the idea that the non-neural ectoderm makes a significant contribution to the pineal organ.

## DISCUSSION

Cellular differentiation in the emerging pineal organ has attracted attention as a model for photoreceptor specification and for asymmetric neurogenesis. However, the earliest steps of pineal organ formation remain relatively unexplored. Whereas classical fate-mapping studies postulated a neural origin of pineal progenitors (Oksche, 1965; Couly and Le Douarin, 1987), we have found here that at least some pineal progenitors originate in the PPR, an area of non-neural ectoderm that flanks the anterior neural plate and gives rise to the cranial placodes: epithelial specialisations that form the sensory organs of the vertebrate head (Graham and Shimeld, 2013; Schlosser, 2014). Thus, the pineal organ appears to be similar to the pituitary gland, another neuroendocrine gland that develops on the opposite (ventral) side of the diencephalon with a non-neural placodal (Rathke's pouch, adenohypophysis, anterior pituitary) and a neural (neurohypophysis, posterior pituitary) contribution (Sánchez-Arrones et al., 2015; Kiecker, 2018). A dual origin of the pineal organ would also be reminiscent of the vertebrate eye that forms from neural (optic vesicle) and placodal (lens) tissue (Graw, 2010). The similarities between non-mammalian pinealocytes and retinal photoreceptors have previously been interpreted as an indicator of a common origin of the pineal organ and the eye (Ekström and Meissl, 2003), and our study lends weight to this concept of ‘the pineal eye’ by suggesting that they are built from the same tissue types: neural and placodal.

*Otx2* is known to be required for both retinal photoreceptor and pinealocyte differentiation in mice, and we have confirmed that there is a requirement for *otx1/2* function in zebrafish pineal development; however, we were also able to show that Otx gene function is required during the earliest steps of pineal specification where it functions by antagonising anti-pineal FGF signalling. *Fgf8* is expressed in the anterior neural ridge/anterior telencephalon of vertebrate embryos (Shimamura and Rubenstein, 1997; Wilson and Houart, 2004) and several Fgf genes are expressed in the midbrain-hindbrain boundary, a structure that is known to function as an organiser of midbrain and anterior hindbrain development (Kiecker and Lumsden, 2012). Thus, it is conceivable that FGF signalling from both these organiser regions determines the anterior and posterior boundaries of the pineal progenitor domain. The timing of the onset of Fgf gene expression (*fgf3* and *fgf17*) at the anterior border of the neural plate is compatible with the hypothesis of FGFs delimiting this progenitor domain anteriorly (Reifers et al., 2000; Raible and Brand, 2001; Shinya et al., 2001; Walshe and Mason, 2003), whereas the gap between the *flh* domain and the anterior border of the *erm* domain in the midbrain appears to be at odds with its posterior border being regulated by FGFs (Fig. 6A,A′). However, it is possible that: (1) different thresholds of FGF signalling activity determine the anterior and posterior borders of this domain; (2) other factors interact with FGFs in this process; and/or (3) cells in this area are differentially competent for anti-pineal FGF activity. The expansion of the pineal anlage in *mbl* mutant embryos suggests that its anterior border is defined by WNT inhibition (Masai et al., 1997) and telencephalic expression of Fgfs is absent in these mutants*,* indicating that WNTs are one such group of signals that interact with FGFs and that FGF activity functions downstream of, or in parallel with, WNT signalling in setting the anterior border of the pineal progenitor domain.

Our study has defined the early steps of pineal specification during gastrulation and has indicated that the pineal organ, like the pituitary gland and the vertebrate eye, may have a dual origin, being derived from both neural and placodal tissue. How strong is the evidence for an involvement of neuroectoderm in pinealogenesis? While classic fate-mapping studies suggested an origin of pineal progenitors in the anterolateral neural plate (Couly and Le Douarin, 1987; Eagleson and Harris, 1990) and morphological studies clearly show that pinealogenesis involves an outpocketing of the roof of the diencephalon (Oksche, 1965), there are currently no genetic fate-mapping or gain or loss-of-function studies that have systematically addressed whether neuroepithelial cells contribute to the pineal organ. Testing the extent of a neural contribution to the pineal organ through a genetic loss-of-function approach is likely to be difficult, as any treatment that results in ablation of the neural plate (such as in Khokha et al., 2005) would inevitably also affect the formation of the neural plate border region.

In order to better understand the entire process of pinealogenesis, we now need to investigate how pineal progenitors are assembled into a functional pineal organ. Our data suggest that the non-neural origin of pineal progenitors is conserved between anamniotes and amniotes; however, there are considerable differences in the pineal bauplan of different species, and it will be an interesting challenge to reconcile conserved genetic with divergent morphogenetic programmes. The mature pineal organ consists of multiple cell types – pinealocytes as well as specialised neurons and glia – and the relative contributions of these differ between different vertebrate species. Thus, our study raises the interesting issue of cell lineage: are the neural and non-neural pineal progenitors equivalent in generating the different cell types of the pineal organ, or are they the precursors of specific subpopulations of pineal cells? More extensive and long-term lineage-tracing experiments in multiple species will be required to address this question.

## MATERIALS AND METHODS

### Zebrafish husbandry

The following zebrafish (*Danio rerio*) lines were used: AB wild type, *mbl^tm13^* (*mbl*^−/−^) (Heisenberg et al., 1996), *noto^n1^* (*flh*) (Talbot et al., 1995), Tg(*her5:eGFP*) (Staudt and Houart, 2007) and Tg(hsp70:*dnfgfr1-IRESeGFP*) (Lee et al., 2005). Zebrafish were maintained at 28°C on a 14 h light/10 h dark cycle. Collected embryos were cultured in fish water containing 0.003% 1-phenyl-2-thiourea to prevent pigmentation and 0.01% methylene blue to prevent fungal growth. The animal experiments have been authorised by the KCL Ethic Review Committee under HO licence 70/7577.

### Whole-mount *in situ* hybridisation and immunohistochemistry

Standard procedures were followed for *in situ* hybridisation analysis using full-length probes (Thomas-Jinu and Houart, 2013). For whole-mount *in situ* hybridisation followed by detection of ectopically expressed GFP-tagged proteins, embryos were incubated in Fast Red substrate solution [buffered in 0.1 M Tris-HCl, 100 mM NaCl (pH 8.2); Roche] for at least 2 h at room temperature (or at 4°C overnight). Immunohistochemistry was performed using rabbit anti-GFP pAb [Torrey Pines Biolabs, AMS Biotechnology (Europe), TP401] or mouse anti-DLX3b (ZIRC; AB_10013771). Embryos were blocked in 10% goat serum/PBST (0.1% Tween-20 in phosphate-buffered saline) and incubated overnight in a dilution of primary antibody (1:500 for anti-GFP, 1:50 for anti-DLX3b) in PBT containing 1% goat serum. Three 10 min washes in PBT were followed by incubation with a 1:500 dilution of secondary antibody coupled to AlexaFluor 488 (Invitrogen; A32723 and A32731). Confocal imaging was performed on a Nikon Eclipse C1 microscope. Images were processed using ImageJ and Adobe software.

### RNA/DNA/MO injections

Capped RNA was transcribed with SP6 RNA polymerase using the mMessage mMachine Kit (Ambion) and injected into zebrafish embryos at one-cell stage. *dlx3b* and *flh/noto* RNA (1 nl) were injected per embryo at 50 ng/µl and 25 ng/µl, respectively. *Kaede* and *H2B-GFP* RNA (1 nl) were injected per embryo at 25 ng/µl each. *hs:dlx4b-GFP* DNA (1 nl of 50 ng/µl) and/or *hs:flh-GFP* DNA (1 nl of 30 ng/µl) were injected at one cell-stage and heat shock was performed at 70% epiboly for 30 min at 37°C. Morpholino antisense oligonucleotides (GeneTools) against *dlx3b*, *dlx4b*, *flh/noto*, *sox10* and *foxd3* were used at a concentration of 2 ng/nl, 1 nl per embryo. Mixed MOs against *otx1* and *otx2* (*otxH*) were injected as described by Foucher et al. (2006).

### Lineage tracing in zebrafish

Cell lineage tracing with fluorescein was performed as described by Staudt and Houart (2007). For time-lapse cell tracking experiments, zebrafish embryos were injected with *Kaede* and *H2B-GFP* RNA at the one-cell stage. At bud stage, cells were photo-converted at 405 nm using a Leica SP5 confocal microscope, and embryos were imaged with a Zeiss Z.1 light-sheet fluorescence microscope for 12 h with a time step of 6 min. Images were processed with the ZEN and Arivis softwares, and cells were tracked manually in ImageJ using a custom-made plugin.

### Chick *in situ* hybridisation

Chicken (*Gallus gallus*) eggs were obtained from Stewart (UK) and incubated at 38°C until embryos developed to desired stages (Hamburger and Hamilton, 1951). Embryos were fixed in 4% paraformaldehyde in PBS at 4°C and *in situ* hybridisation was performed as previously described using probes for *FGF8*, *NOT1* (a kind gift from M. Kessel, Georg-August-Universitaet Goettingen, Germany) and *SOX2* (Chapman et al., 2002).

### *In ovo* electroporation and GFP immunohistochemistry

Chick eggs were incubated for 25-29 h at 38°C until embryos reached the desired stages. Eggs were windowed, a small amount of Pelikan Fount India ink (diluted 1:5 with Tyrode's solution) was injected beneath the embryo into the yolk sac to increase the embryo's visibility, the vitelline membrane was locally removed, and the plasmids pEx-FGF8 and pEx-dnFGFR, respectively (a kind gift from E. Grove, The University of Chicago, IL, USA), were injected together with pCAβ-eGFP and Fast Green (1 mg/ml for each plasmid) into the anterior neural tube or between the anterior neural folds of the embryo. Two platinum-iridium electrodes were placed on either side of the embryo and four 10 V pulses of 20 ms were supplied to transfect the right side of the neural folds/tube. Eggs were re-sealed and incubated for another 24-48 h before dissection and fixation. Only embryos that had a distinct recognisable diencephalic vesicle and did not show gross overall brain abnormalities were selected for analysis. Post *in situ* hybridisation staining for GFP was performed using rabbit anti-GFP antiserum at a concentration of 1:200 (Invitrogen, ThermoFisher; A-6455) followed by incubation with a 1:200 dilution of secondary antibody coupled to Alexa Fluor 488/568 (Invitrogen; A32731 and A-11011)

## Supplementary Material

Supplementary information
