# Supplementary material for: Pineal progenitors originate from a non-neural territory limited by FGF signalling
Source: Development. 2019 Nov 21;146(22):dev171405. doi: 10.1242/dev.171405 (PMC7375831; doi:10.1242/dev.171405)
Supplement: Supplementary information [file develop-146-171405-s1.pdf]

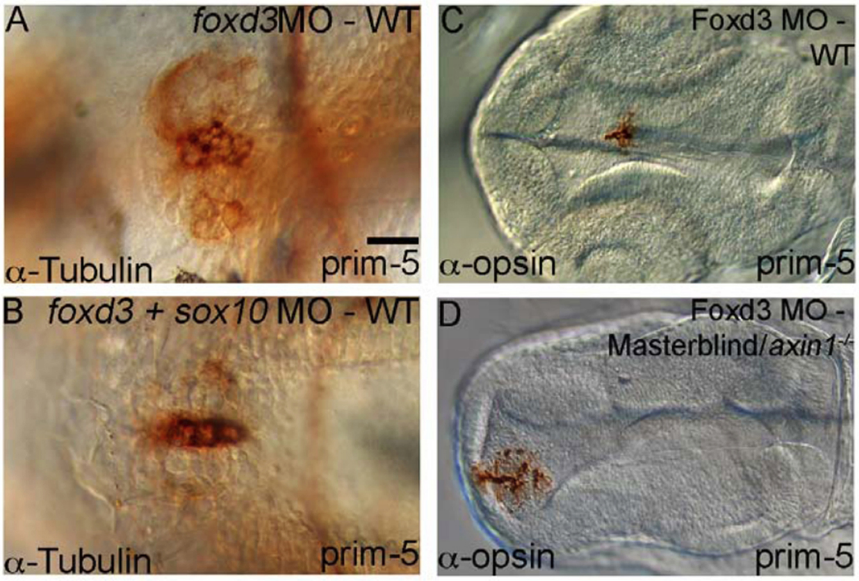

Fig. S1. Neural crest identity is not required for pineal organ formation and the pineal anlage is expanded in embryos with excessive Wnt signalling.
Dorsal views (anterior points left) of zebrafish embryos at prim-5 stage stained immunohistochemically for the neural differentiation marker α-Tubulin (A, B) and the photoreceptor marker α-Opsin (C, D). Neither MO-mediated knockdown of *foxd3* alone (A, C), nor of *foxd3* and *sox10* together (B) affects pineal organ formation. (D) The pineal anlage is anteriorly expanded in *mbl* mutants. Scale bar in A is 50 μm.

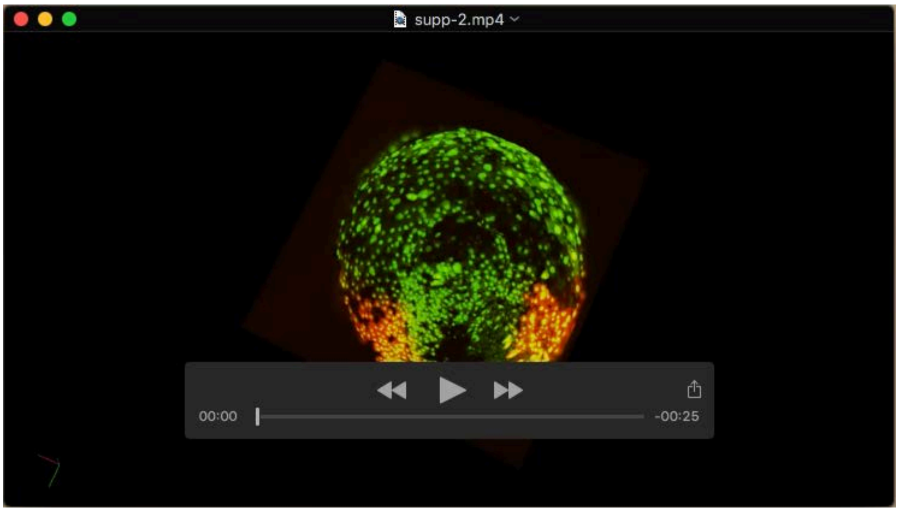

Movie 1. Non-neural ectoderm contributes to the pineal organ. Light-sheet microscopy movie of zebrafish embryo (dorsal view, anterior to the top) expressing Kaede from tail bud to 22-somite stage (T step: 6 min). Photo-converted cells located in the non-neural ectoderm end up in the pineal organ. Also see Fig. 2G-J.

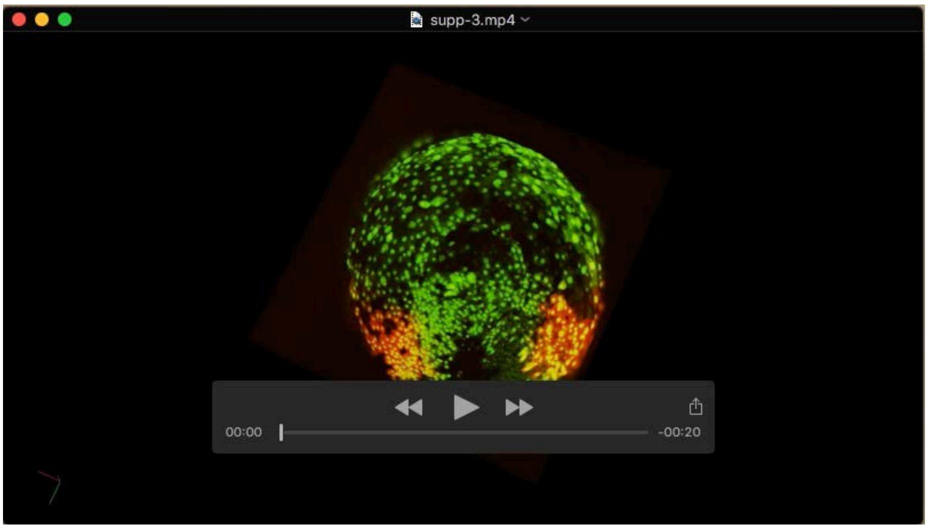

Movie 2. Tracks of non-neural ectodermal cells ending up in the pineal organ. Tracks of three converted cells located in the non-neural ectoderm at tail bud stage that end up in the pineal organ at 30 hpf. Also see Fig. 2G-K.
